# Increasing riparian vegetation cover to improve water quality: the importance of considering land use

**DOI:** 10.1007/s00267-026-02478-1

**Published:** 2026-04-27

**Authors:** Payton A. J. Te Ngaio, Joseph M. McMahon, Deanna van den Berg, Al Healy, Ryan D. R. Turner, Angela Marsh

**Affiliations:** 1 Reef Catchments Science Partnership, School of the Environment, The University of Queensland, Brisbane, Australia; 2 Queensland Department of the Environment, Tourism, Science and Innovation Australia, Brisbane, Australia

**Keywords:** Watershed, Sediment, Remote sensing, Catchment management, Sugarcane cropping, Riparian cover

## Abstract

Riparian vegetation plays a crucial role in regulating sediment dynamics, reducing surface runoff, mobilising sediment and stabilising streambanks. Despite extensive research on sediment loads and riparian vegetation individually, there remains a gap in understanding their interrelationship, particularly within the context of water quality and catchment management. This study investigates the statistical association between water quality and riparian vegetation cover within the Herbert catchment, Far North Queensland, Australia. Over one million total suspended sediment equivalent (TSSeq) data points were collected from 14 monitoring sites between December 2020 and December 2023, averaged into 361 monthly samples and paired with site-specific total cover (TC) values. Using Spearman’s rank correlation across land use disturbance classes (minimal, moderate, high) and seasonal subsets, results revealed a significant overall negative correlation between TSSeq and TC (*ρ* = –0.431, *p* < 0.0001). The strength of this relationship declined with increasing disturbance: minimal disturbance sites showed the strongest correlation (*ρ* = –0.530, *p* < 0.0001), while at high disturbance sites the correlation was not significant (*ρ* = 0.075, *p* > 0.05). Seasonal analysis showed stronger correlations during the wet season, except in high disturbance areas, where the dry season correlation was higher but still not statistically significant. Limitations in TC’s ability to distinguish vegetation types and capture dynamic cover changes in disturbed areas are discussed. These findings highlight the importance of riparian vegetation in improving water quality and underscore the need for refined remote sensing methods when integrating high-resolution temporal water quality datasets.

## Introduction

The protection and rehabilitation of riparian zones is frequently advocated to balance land use pressures and in-stream water quality (Alemu et al., 2017; Dosskey et al., 2010; Gomes et al., 2014; McKergow et al., 2004). Riparian restoration is globally recognised as an effective strategy for improving land and water quality by enhancing channel stability, reducing erosion and reducing nutrient runoff (González et al., 2015; Hamman et al., 2022; Singh et al., 2021). Riparian vegetation is critical to maintain essential ecosystem functions such as regulating sediment and nutrient transport (Fernandes et al., 2011; Fernández et al., 2014). The nature of riparian vegetation cover can result in varying ecosystem benefits specific to the landscape, such as channel stabilisation by large woody vegetation or surface runoff filtration by dense herbaceous vegetation (Alemu et al., 2017; Dosskey et al., 2010; McKergow et al., 2004). Studies have demonstrated that reduced riparian vegetation cover has led to increased rates of erosion (Bartley et al., 2008; McKergow et al., 2004). However, the extent of the impact on water quality depends on a range of hydrologic, climatic and biological factors like soil type and slope, vegetation structure and species composition (Dosskey et al., 2010). Understanding the environmental conditions unique to a given location enhances our ability to identify factors that influence the role of riparian vegetation in delivering water quality outcomes. This is critical for targeted restoration efforts, tailored to local ecological and hydrological conditions.

In the catchments of the Great Barrier Reef (GBR), Australia, protection and rehabilitation of riparian vegetation forms a key component of strategies to reduce diffuse water pollution (Waterhouse et al., 2024). Numerous studies in the Great Barrier Reef catchment area (GBRCA) have been used to inform policy and guide improvement initiatives surrounding ongoing environmental pressures (Brodie et al., 2009; Waterhouse et al., 2024). Current estimates suggest that fine sediment export to the GBR is approximately 1.4 to 5 times greater than pre-development loads (Prosser and Wilkinson, 2024). Vegetation degradation through land/tree clearing, changes in the structure and function of grass species and low ground cover are amongst key drivers of anthropogenic sediment from the GBRCA (Wilkinson et al., 2024). Despite ongoing efforts, current land management strategies have yet to fully combat the impact of historic landscape modification and subsequent environmental decline (Eberhard et al., 2017; Hamman et al., 2022; Lawson et al., 2007).

Despite its widespread recommendation in policy documents, there has been limited research on water quality responses to varying riparian vegetation extents between sites at a catchment scale (Feld et al., 2018; Lorenz and Dosskey et al., 2010; Feld, 2013). Olley et al. (2015) is one of the few studies to explore this within Australia and highlighted a significant relationship between total suspended sediment (TSS) load and the proportion of remnant vegetation^1^ in Southeast Queensland during high flow events. The paper estimated catchments containing no remnant vegetation produced 50 to 200 times more sediment per unit area than those with fully vegetated channels. These findings suggest there may be a correlation between sediment loads and riparian vegetation within GBR catchments.

Water quality monitoring is commonly used to identify priority areas for intervention and indicate environmental improvement. High-frequency in situ sensors are increasingly employed for water quality monitoring due to their ability to capture fine-scale temporal variations, including event-driven processes (Rode et al., 2016; Rozemeijer et al., 2025).

The aim of this study was to investigate the relationship between water quality and riparian vegetation cover using total suspended sediment equivalents (TSSeq) data collected in the Herbert River catchment within the GBRCA. This dataset was paired with monthly spatially estimated measures of total cover in the riparian zone. The three primary research objectives were to (1) analyse the temporal interaction between water quality and riparian vegetation cover to understand the seasonal effect between the wet and dry season, (2) assess the spatial variation between water quality and riparian vegetation cover to understand the influence of dominant land use (cropping, conservation and grazing native vegetation) on water quality and riparian vegetation and (3) confirm whether the riparian zone influences water quality.

## Methods and materials

### Study Area and Sampling Sites

**Fig. 1:**
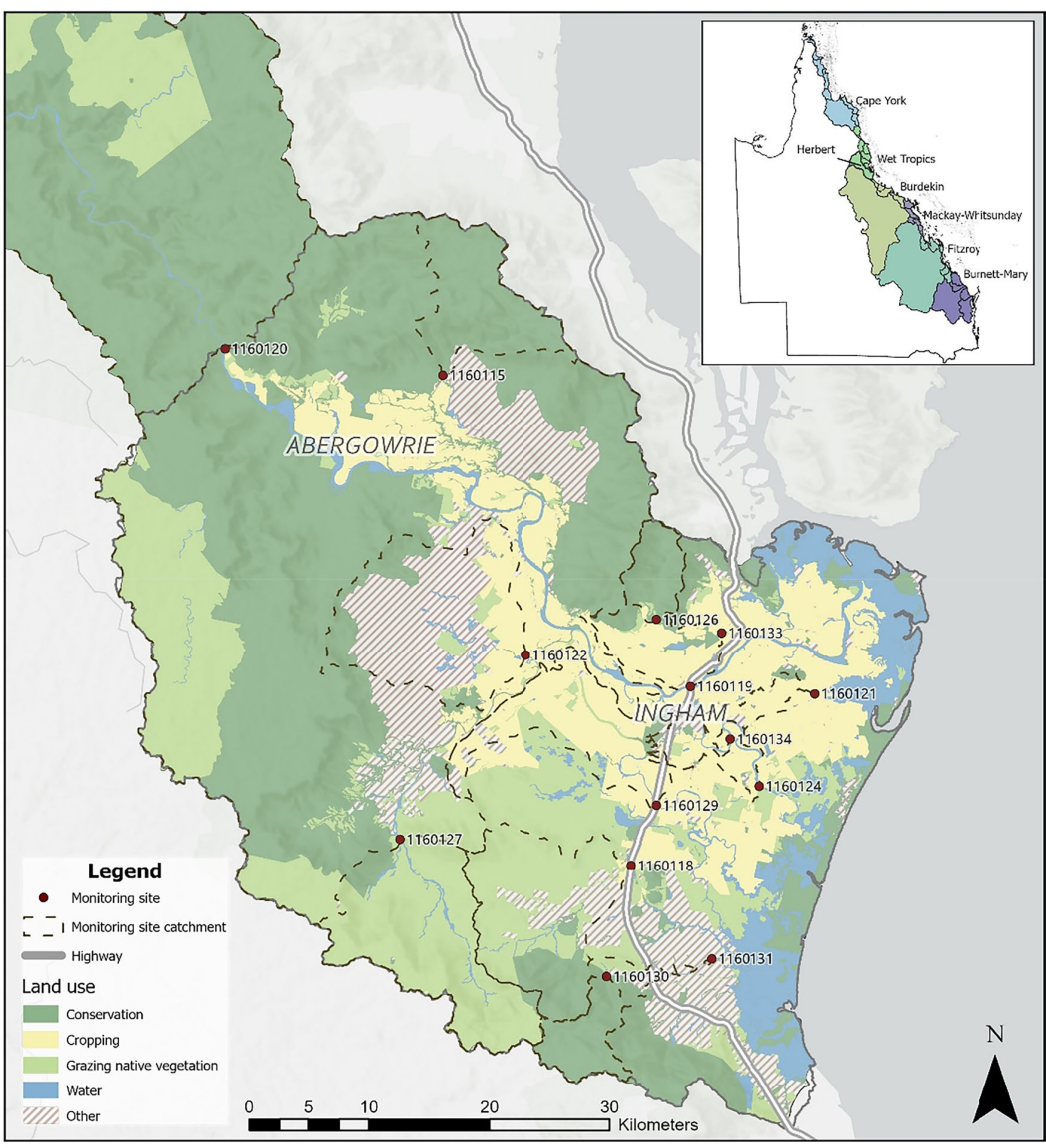
Map of lower Herbert catchment, located within the Ingham region, Queensland, showing the Herbert water quality monitoring sites, catchment areas and dominant land uses. Conservation (green), cropping (yellow) and grazing native vegetation (light green) areas are highlighted, along with highways (grey lines) and water bodies (blue). Red dots indicate monitoring sites, with site codes labelled and site catchment areas outlined with black dashes. The insert map shows the location of the study area within Queensland and the Great Barrier Reef catchment Natural Resource Management regions

The Herbert catchment is located in far north Queensland and is the largest catchment in the Wet Tropics region (9842 km^2^) (State of Queensland, 2018b). Rainfall and groundwater inputs to the Herbert basin are variable throughout the year and can experience several high flow or flood periods during the wet season (Australia Bureau of Meteorology et al., 2019). The Herbert River meanders through three distinct sections characterised by differing land uses and physiography (Fig. 1)(Bartley et al., 2003; Johnson et al., 2000). The upper section is dominated by cattle grazing with minor areas of land for horticulture and dairy, the middle section is protected Wet Tropics World Heritage Area and state forest and the lower section has extensive sugarcane cropping along with areas of conservation and grazed native vegetation.

The Herbert catchment has lost the largest proportion of riparian woody vegetation since pre-European settlement among Wet Tropics catchments (State of Queensland, 2019). Streambank erosion is the primary contributor of sediment in the catchment, followed by hillslope erosion (McCloskey et al., 2021; Tims et al., 2010). Erosion rates are greater in areas with high land use intensity, particularly in regions dominated by grazing and sugarcane cultivation (Tims et al., 2010).

The Herbert is listed as one of the top three priority catchments for sediment reduction in the GBRCA and has extensive water quality monitoring in the lower section (hereafter referred to as the lower Herbert) (State of Queensland, 2018a). Each monitoring site is positioned at a singular point to capture upstream pollutant sources. The monitoring program uses topography to delineate the boundaries and shape of a monitoring site’s catchment area by defining the direction of surface water flow and the area upstream of the monitoring site point that contributes to runoff.

### Sampling Design And Processing

Fourteen monitoring sites in the lower Herbert water quality monitoring program were selected to collate data on total suspended sediment equivalents (TSSeq) from December 2020 to December 2023. Samples are collected using TriOS^TM^ Opus spectral sensors and transmitted hourly to the Department of the Environment, Tourism, Science and Innovation (DETSI) Water Quality Investigations online data portal, eagle.io. The data was processed through an automated data quality assurance and control pipeline adapted from a proposed framework by Leigh et al. (2019). Data was periodically reviewed to validate automated outputs, identify significant deviations and flag values for additional assessment and site-specific analysis. In total, over 1 million data samples were collated and averaged to monthly estimates. The sample periods and counts for each site are detailed in Table 1.

**Table 1:** Information summary of sites selected from the Herbert catchment water quality monitoring sites, including sub catchment, site ID, site name, latitude, longitude, site area (km^2^), riparian area (km^2^), number of samples collected and the sampling period

Sub Catchment	Site ID	Site name	Latitude	Longitude	Site catchment area (km^2^)	Riparian area (km^2^)	Number of months sampled
Herbert River	1160115	Broadwater Creek at Day Use	–18.41633	145.94393	90	17	27
Francis Creek	1160118	Francis Creek at Weir	–18.76673	146.13407	143	31	21
Herbert River	1160119	Herbert River at John Row Bridge	–18.62831	146.16486	8608	1575	21
Herbert River	1160120	Herbert River at Nash’s Crossing	–18.41435	145.77097	6739	1191	25
Victoria Creek	1160121	Lagoon Creek at Five Mile Road	–18.62341	146.2632	18	4	24
Lannercost Creek	1160122	Lannercost Creek at Lannercost Ext Road	–18.6185	146.03286	180	26	21
Palm Creek	1160124	Palm Creek at Bosworths Road	–18.69688	146.22796	32	2	29
Ripple Creek	1160126	Ripple Creek at Gangemis Road	–18.5811	146.1323	31	6	32
Stone River	1160127	Stone River at Running Creek	–18.76607	145.95028	164	39	26
Palm Creek	1160129	Trebonne Creek at Bruce Highway	–18.7197	146.14842	95	9	36
Cattle Creek	1160130	Waterview Creek at Jourama Road	–18.85141	146.12466	38	5	27
Cattle Creek	1160131	Waterview Creek at Mammarellas Road	–18.82947	146.20546	53	9	23
Herbert River	1160133	Ripple Creek at Seymour Creek Gates	–18.58571	146.18459	83	9	26
Palm Creek	1160134	Palm Creek at Cemetery Road	–18.6643	146.2006	17	2	26

### Riparian Buffer Data and Processing

A total riparian vegetation cover (TC) metric was derived using the monthly blended fractional cover dataset produced by the Joint Remote Sensing Research Program and Queensland Government’s DETSI Ground Cover Monitoring program (Joint Remote Sensing Research et al., 2022). This dataset consists of medoid-composited monthly fractional cover created from a combined Landsat 8 and 9 and Sentinel-2 time series, with at least three cloud free observations of fractional cover imagery required for a representative pixel to be included in the monthly composite. Riparian buffers were defined as 50 m around waterways above stream order 1 to align with the Paddock to Reef monitoring methodology (Australia and Queensland governments, 2024). The calculated riparian area within the contributing catchment area of each monitoring site is provided in Table 1. Using ArcGIS Pro, we extracted the mean percentage of green vegetation (e.g. leaves, grass and growing crops), non-green vegetation (e.g. branches, dry grass, hay and dead leaf litter) and bare ground (soil or rock) within riparian buffers for catchments draining to each monitoring site. The green and non-green fractions were combined to create the TC metric. The metric provides an estimated percentage of riparian buffer covered by vegetation and represents a combination woody and non-woody vegetation without distinguishing between vegetation type or condition.

The riparian buffers for catchments draining to each monitoring site were also used to extract the percent of land used for conservation, cropping and grazing native vegetation, woody vegetation extent, slope steepness (S) factor and soil erodibility (K) factor. Land use percentages were calculated using the GBR land use mapping 2021 dataset. Land use classes are based on a modified Australian Land Use Management Classification (ALUMC) Schema, Version 9 (October 2016) (State of Queensland, 2023). The presence of woody vegetation was determined through the use of the Statewide Landcover and Trees Study (SLATS) woody vegetation extent dataset, where stands of woody vegetation greater than 0.5 ha with a canopy density greater than 10% crown cover are included (State of Queensland, 2024). The K factor and S factor were selected as the two most relevant factors in the revised universal soil loss equation to inform spatial differences in soil erosion potential across the study area. The K factor is derived from soil data in the Australian Soil Resource Information System (State of Queensland, 2016a) and the S factor is derived from a Digital Elevation Model created by the Shuttle Radar Topography Mission (State of Queensland, 2016b). Table S1 outlines the riparian buffer characteristics identified within the contributing catchment area for each monitoring site and incorporated into the data analysis.

### Data Analysis

All available TSSeq monitoring data between December 2020 and December 2023 was included in the analysis, with the sampling date and total number of samples varying between sites. The total months of data for each site are provided in Table 1.

A Principal Component Analysis (PCA) was undertaken to explore the variables with the highest contribution to site variability. The analysis included averaged annual wet season (November to March) and dry season (April to October) TC and TSSeq samples and incorporated riparian buffer characteristics outline in Table S1. This ensured the PCA reflected spatial differences and minimised the contribution of temporal variability in monthly TC and TSSeq estimates. Prior to analysis, all variables were standardised by subtracting the mean and dividing by the standard deviation, resulting in values with a mean of 0 and a standard deviation of 1. Sites were then plotted along the first two principal components to reveal clustering patterns and define groups. A biplot was utilised to understand how variables responded to each other and corresponded to the sites and identified clusters in the PCA.

The statistical relationship between TSSeq and TC was evaluated using paired samples. Initial data exploration indicated non-normal distributions for both variables. Consequently, Spearman’s ranks correlation was selected as a non-parametric method to assess the strength and direction of the association between TSSeq and TC. Statistical significance was determined at a threshold of *p* < 0.05. Correlation analysis was conducted on the entire dataset and separated by wet and dry season, to estimate the influence of temporal variation. Linearity was visually examined through a bivariate linear regression graph.

All statistical analyses were conducted using the R 4.4.2 Hmisc package (R Core Team, 2016).

## Results and discussion

This study aimed to examine the statistical relationship between water quality (measured TSSeq) and riparian vegetation cover (measured total cover (TC)) within the Herbert catchment. The analysis of correlations between TC and TSSeq revealed seasonal and grouped patterns reflective of the spatial distribution of riparian characteristics that highlight the role of riparian vegetation in sediment retention across varying land-use conditions. Here we highlight the importance of maintaining high functioning riparian zones for water quality improvements and the effect of land use disturbance. Our findings provide valuable insight to support evidence-based riparian management practices and contribute to the development of more effective catchment scale environmental policies.

### Cluster Analysis

**Fig. 2:**
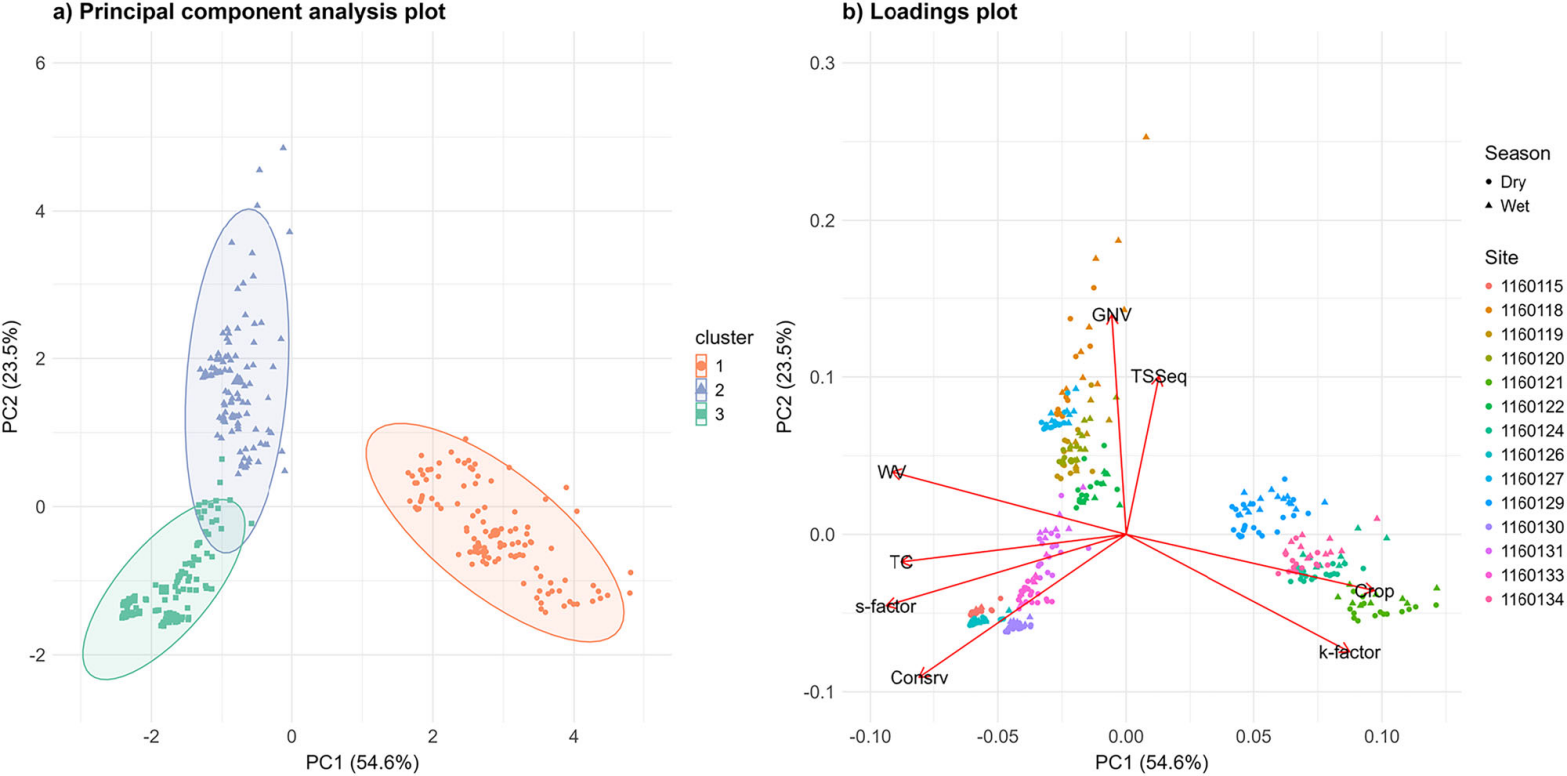
Principal component analysis (PCA) of site-level riparian buffer conditions. **a** PCA ordination plot showing the distribution of sites across the first two principal components (PC1 and PC2), coloured by cluster assignment. Ellipses represent the 95% confidence region for each cluster. **b** Corresponding loadings plot displaying the contribution and direction of environmental variables used to define clusters, including, percent woody vegetation (WV), total riparian vegetation cover (TC), mean monthly total suspended sediment equivalents (TSSeq), slope stability (s-factor), soil erodibility (k-factor) and percent land used for grazing native vegetation (GNV), conservation (Consrv) and cropping (Crop). Vector lengths indicated the strength of each variable’s influence, with points coloured by site and shape by season (Dry or Wet)

The PCA cluster (Fig. 2a) and corresponding biplot (Fig. 2b) was employed to differentiate sites using TC, TSSeq and variables outlined in Table S1. The ordination patterns indicate the presence of three clusters, with separation primarily driven by differences in dominant land use type (grazing native vegetation, conservation and cropping).

The first and second principal components (PC1 and PC2) explained 54.6% and 23.5% of the total variability, respectively. The first principal component was primarily associated with a strong positive association for cropping (0.44) and k-factor (0.40), and a strong negative association for s-factor (–0.43), woody vegetation (–0.41), TC (–0.40) and conservation (–0.37). The second principal component was primarily associated with a strong positive association for grazing native vegetation (0.63). Compared with the other principal components, the third principal component accounted for 10.45% of the total variance and was dominated by TSSeq (0.83), with only minor contributions from the other variables (<0.30). This indicates that the third axis primarily reflects a TSSeq gradient largely independent of the land use contrasts resolved by PC1 and PC2.

Within Fig. 2a, sites in cluster 1 (1160121, 1160124, 1160129 and 1160134, as identified by Fig. 2b) are distinct due to their strong association with high cropping intensity and elevated k-factor. In contrast, the overlap between cluster 1 and 3 in Fig. 2a and the position of woody vegetation in Fig. 2b indicate that sites within these two groups share similar levels of woody vegetation cover. Cropping and woody vegetation show an inverse relationship, which is also observed, though less strongly, between cropping and TC. However, TC is more closely related to conservation land use, suggesting that these sites may have a higher diversity of vegetation cover types beyond woody vegetation.

### Site Classification

Based on the findings presented in Fig. 2a, b, the percentage of woody vegetation cover and land used for grazing native vegetation, conservation and cropping was identified as the main separator between the majority of sites. Comparatively, the s-factor and k-factor further differentiated the sites due to variation in topography and land condition. Table 2 lists each site and their site classification which is defined as; (1) Minimal disturbance—site dominated by conservation land use or grazing present but < 10% (approx.) of site and with > 90% woody vegetation cover, (2) Moderate disturbance—sites dominated by grazing, forestry or conservation present but < 35% (approx.) with > 90% woody vegetation cover, (3) High disturbance—site dominated by cropping land use.

**Table 2:** Sites and defined site class

Site	Site class
1160115	Minimal disturbance
1160118	Moderate disturbance
1160119	Moderate disturbance
1160120	Moderate disturbance
1160121	High disturbance
1160122	Moderate disturbance
1160124	High disturbance
1160126	Minimal disturbance
1160127	Moderate disturbance
1160129	High disturbance
1160130	Minimal disturbance
1160131	Minimal disturbance
1160133	Minimal disturbance
1160134	High disturbance

In addition to containing high proportions of cropping land use, the high disturbance sites (1160121, 1160124, 1160129 and 1160134) were distinguished by notably lower s-factor values and elevated k-factor values. Whereas, low and moderate disturbance sites exhibited comparable proportions of woody vegetation cover, cropping and k-factor values. The moderate disturbance sites (1160118, 1160119, 1160120, 1160122, 1160127) demonstrated more heterogeneous land use patterns, with a greater mix between conservation and grazing of native vegetation. In contrast, the minimal disturbance sites (1160115, 1160126, 1160130, 1160131, 1160133) were characterised by similar proportions of conservation land use and woody vegetation cover.

### Correlation Analysis

At the catchment scale, our results were consistent with those reported by Olley et al. (2015). In the Herbert catchment, TSSeq concentrations (i.e. water quality improves) were negatively correlated with total riparian vegetation cover increases (*ρ* = –0.431, *p* < 0.0001), indicating that higher vegetation cover was generally associated with lower suspended sediment levels across seasons and disturbance categories. As land use disturbance increased, the correlation strength and the direction varied, ranging from a strong negative association under minimal disturbance (*ρ* = –0.530, *p* < 0.0001) to a weak and non-significant association under high disturbance (*ρ* = 0.075, *p* > 0.05).

**Fig. 3:**
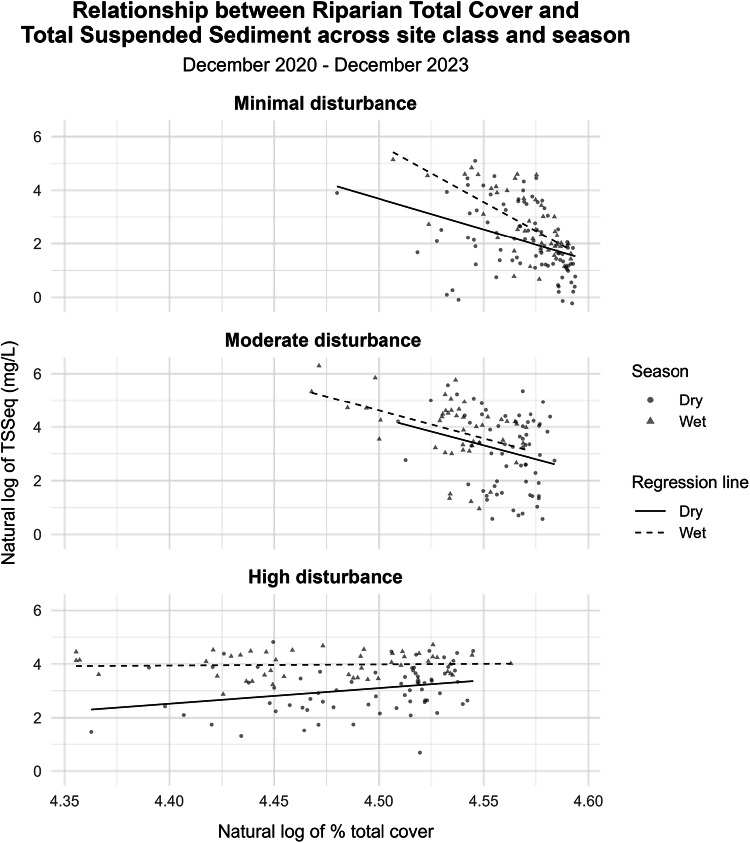
Natural log of monthly mean total suspended sediment equivalents (TSSeq) against natural log of total riparian vegetation cover (%) between December 2020 to December 2023 with point shapes and regression line type representing season (wet and dry). The solid line and circle dots represent dry season samples and the dash and triangle points represent wet season samples. Colours correspond to site classification of either high disturbance (red), minimal disturbance (green) or moderate disturbance (blue)

Figure 3 illustrates the distribution of TSSeq in relation to TC across site classification and seasonal periods. Each data point represents a monthly observation, with marker shapes distinguishing between wet season (November to March) and dry season (April to October) samples. Seasonal differences in TSSeq are evident, with higher concentrations recorded during the wet season across all disturbance groups.

The moderate disturbance group recorded the average highest TSSeq values. These seasonal differences coincide with periods of increased rainfall and surface runoff, which commonly enhances erosion potential and sediment transport in tropical catchments.

Across disturbance groups, the minimal disturbance sites consistently exhibited higher vegetation cover, while the moderate and high disturbance sites showed greater seasonal variability. As shown in Table 3, the negative correlation between TSSeq and TC was strongest during the wet season, suggesting that the association between vegetation cover is more high-flow conditions. During the dry season, when discharge and erosion-related processes are reduced, TSSeq concentrations were generally lower, and the correlation between TSSeq and TC was weaker across most disturbance classes.

**Table 3:** Summary of Spearman’s rank correlation coefficient (ρ) between Total Cover (TC) and total suspended sediment (TSSeq) within each site class and between wet and dry seasons

		TSSeq		
Site class	TC ~	Mean	Mean - Wet	Mean - Dry
Minimal disturbance	*ρ*	**–0.530**	**–0.668**	**–0.474**
	*p-value*	**<0.0001**	**<0.0001**	**<0.0001**
Moderate disturbance	*ρ*	**–0.390**	**–0.443**	**–0.208**
	*p-value*	**<0.0001**	**<0.01**	**<0.001**
High disturbance	*ρ*	0.075	0.039	0.305
	*p-value*	0.429	0.795	**0.011**
All sites combined	*ρ*	**–0.431**	**–0.546**	**–0.326**
	*p-value*	**<0.0001**	**<0.0001**	**<0.0001**

Values in bold are significant at the 0.05 level

Among the three site classifications, the minimal disturbance group exhibited the strongest negative correlation between TSSeq and TC (*ρ* = –0.530, *p* < 0.0001). These sites were characterised by greater proportions of conservation land use and woody vegetation. The moderate disturbance group also showed a significant negative correlation in both the combined dataset (*ρ* = –0.390, *p* < 0.0001) and wet season subset (*ρ* = –0.443, *p* < 0.01), although to a lesser degree than the minimal disturbance sites. In contrast, the high disturbance group displayed no significant correlation overall (*ρ* = 0.075, *p* = 0.429). Within this group, only the dry season subset showed a statistically significant correlation (*ρ* = 0.305, *p* = 0.011), while the wet season correlation was weak and not significant (*ρ* = 0.039, *p* = 0.795).

The results indicate that in regions experiencing minimal to moderate land use disturbance the negative correlation between TSSeq and TC is more apparent. The minimal disturbance sites, which were predominantly located within conservation areas and exhibited high TC values, likely represent areas high in remnant vegetation. These findings are consistent with those of Olley et al. (2015) who identified a statistically significant relationship between TSS load and the proportion of remnant vegetation in Southeast Queensland. Similar land use observations were observed in de Mello et al. (2018) who found a negative correlation between forest cover and TSS and organic suspended solids concentrations, alongside a positive correlation between agricultural land use and elevated levels of the sediment indicators in Southeastern Brazil. In comparison, our results suggest that in highly disturbed areas, associations between TSSeq and TC are less evident, potentially reflecting the influence of additional sediment generating processes that may obscure vegetation-sediment relationships or inherent limitations in the temporal resolution of TC.

The monthly fractional cover dataset, created from a combined Landsat 8 and 9 and Sentinel-2 time series, predicts vegetation cover at medium spatial resolution (30 m). In areas with minimal disturbance, vegetation cover is expected to remain relatively consistent within the monthly time period and the observations used to create the composite image are likely to be relatively similar. The vegetation cover in highly disturbed agricultural landscapes is more variable, due to the dynamic nature of cropping systems. The observations used for a monthly composite may include abrupt changes due to harvest or planting for example, with the change in cover more rapid than the monthly time scale. Changes in cropping areas may also be missed due to cloud cover and therefore not represented in the monthly dataset. The spatial arrangement of cover in highly disturbed areas is also more variable than in areas with minimal disturbance. Cropping areas with high cover may still present with persistently bare inter-rows, for example, contributing to substantial surface runoff during rainfall events.

In addition, scale mismatches and mixed-pixel effects from adopting a 50 m buffer and utilising a remote sensing product with 30 m spatial resolution were considered as a limitation, particularly for accurately capturing TC. Methods that improve the spatial resolution to 10 m may better represent TC in both minimal to high disturbance landscape, however 10 m factional cover datasets are not currently publicly available as a monthly product. While single date fractional cover imager can be produced, analysis of these datasets was out of scope for this study. Comparable limitations associated with remote sensing approaches are noted by Allred et al. (2025) and Saldarriaga (2022) who highlight challenges specific related to unique vegetation patterns and cover type in riparian areas. Saldarriaga (2022) suggests that while Sentinel-2 is well suited to broad-scale analyses, fine-scale features such as riparian buffers may require UAV platforms to provide more detailed data, especially when analysing small areas with mixed land use and vegetation types. Similarly, Allred et al. (2025) provides a similar perspective with consideration to understorey vegetation, which may not be well represented, particularly in areas with dense overstory canopies. Nevertheless, both studies emphasise these issues in relation to characterising vegetation attributes such as structure and diversity and to understanding spatial scaling effects, whereas our study specifically focuses on riparian cover and its association with water quality. Balancing the optimal spatial resolution for quantifying vegetation within fine‑scale riparian buffers against the temporal resolution required to detect changes and responses in water quality remains a complex and largely unresolved challenge.

The relationship between TC and TSSeq within high disturbance sites warrants further investigation to clarify the nature of our findings. In landscapes dominated by intensive cropping, enhanced approaches for distinguishing vegetation types within riparian buffers, alongside more precise temporal measures of vegetation cover change following land management practices, may offer deeper insights into water quality responses. Such improvements could strengthen the interpretation of TC across disturbance classes and support more robust comparisons between site classes.

Our findings indicate that areas with high woody vegetation cover, subject to minimal land use disturbance, show reduced in-stream sediment loads. These results support policy direction to increase woody riparian vegetation cover to improve stream water quality. However, as land use disturbance begins to transition from minimal to high, strategies to improve water quality benefits should allow for a temporal lag in water quality improvement until a substantial woody vegetation corridor is established. Long-term monitoring would provide greater insight into the full benefit of riparian restoration efforts and help to quantify the level and duration of riparian restoration needed to deliver measurable water quality improvement. Many riparian management studies are confined to short-term observations (Feld et al., 2018; Muller et al., 2016), that limit the identification of the lag between the time of management intervention and environmental outcome. Whilst the magnitude of lag time can vary between sites and pollutant type (Meals et al., 2010; Swanson et al., 2017), increasing data resolution to evaluate water quality before and after riparian management intervention could improve lag identification and provide insight into improved intervention approaches (Feld et al., 2018; Muller et al., 2016).

## Conclusion

Riparian vegetation plays a critical role in regulating in-stream sediment loads by reducing runoff, trapping sediments and stabilising banks. This study found a significant relationship between total suspended sediment equivalents (TSSeq) and total riparian cover (TC) across 14 sites in the Herbert catchment, particularly during the wet season. However, this relationship weakened with increasing disturbance, due to the influence of land use variability and seasonal cropping dynamics. These findings highlight the importance of riparian vegetation restoration for sediment control, water quality improvements and the need for careful interpretation of remotely sensed-derived vegetative cover information in highly disturbed catchments.

Our results highlight the importance of maintaining high riparian (woody) vegetation cover. In addition, river restoration approaches (improving riparian vegetation cover to improve water quality) need to account for riparian zone fragmentation and its relationship to measurable water quality improvements. Future research should investigate the dominant sediment delivery processes associated with specific land uses within riparian buffers, both at local scales and across the broader catchment. While cropping remains the dominant high disturbance land use in the lower Herbert riparian zone, policy interventions should prioritise the restoration of riparian zones in a systematic and connected approach with a focus on restoring woody vegetation.

## Supplementary information

Supplementary material

## Data Availability

No datasets were generated or analysed during the current study.

## Footnotes

1 Woody vegetation classified as remnant under the *Vegetation Management Act* 1999 when the dominant canopy retains more than 70% of its original height and over 50% of its original cover and primarily consists of species typical of an undisturbed canopy layer. Neldner, V. J., Wilson, B. A., Dillewaard, H. A., Ryan, T. S., Butler, D. W., McDonald, W. J. F., Richter, D., Addicott, E. P., & Appelman, C. N. (2023). *Methodology for survey and mapping of regional ecosystems and vegetation communities in Queensland*. (Version 7.0). Queensland Herbarium, Queensland Department of Environment, Tourism, Science and Innovation, Brisbane.
