# Correction: Increasing riparian vegetation cover to improve water quality: the importance of considering land use

**DOI:** 10.1007/s00267-026-02563-5

**Published:** 2026-07-27

**Authors:** Payton A. J. Te Ngaio, Joseph M. McMahon, Deanna van den Berg, Al Healy, Ryan D. R. Turner, Angela Marsh

**Affiliations:** 1 Reef Catchments Science Partnership, School of the Environment, The University of Queensland, Brisbane, QLD Australia; 2 Queensland Department of the Environment, Tourism, Science and Innovation Australia, Brisbane, QLD Australia

Correction to: *Environmental Management*

https://doi.org/10.1007/s00267-026-02478-1

In this article, the author name Joseph M. McMahon was incorrectly written as Joesph M. McMahon. The original article has been corrected.

