# Supplementary material for: Increasing riparian vegetation cover to improve water quality: the importance of considering land use
Source: Environ Manage. 2026 Apr 27;76(5):174. doi: 10.1007/s00267-026-02478-1 (PMC13121577; doi:10.1007/s00267-026-02478-1)
Supplement: Supplementary file 1 — Supplementary material [file 267_2026_2478_MOESM1_ESM.docx]

# 6.0 Supplementary material

*Table S1 Summary of land use composition, woody vegetation extent, slope steepness (s-factor) and soil erodibility (k-factor) within the riparian buffer for each study site selected from the Herbert Water Quality Monitoring Program.*

| **Site number** | **Conservation (%)** | **Cropping (%)** | **Grazing native vegetation (%)** | **Woody vegetation (%)** | **Slope steepness factor** | **Soil erodibility factor** |
| --- | --- | --- | --- | --- | --- | --- |
| 1160115 | 97.29 | <0.01 | <0.01 | 99.96 | 2.95 | 0.05 |
| 1160118 | 17.09 | 0.30 | 72.50 | 99.28 | 1.15 | 0.04 |
| 1160119 | 35.16 | 1.15 | 46.28 | 94.44 | 1.12 | 0.05 |
| 1160120 | 28.52 | 0.02 | 54.20 | 95.54 | 0.93 | 0.05 |
| 1160121 | 1.05 | 93.39 | 1.14 | 19.02 | 0.05 | 0.07 |
| 1160122 | 19.83 | 4.75 | 5.13 | 90.55 | 0.90 | 0.05 |
| 1160124 | 8.38 | 48.93 | 16.07 | 22.76 | 0.06 | 0.07 |
| 1160126 | 97.76 | 0.13 | 2.11 | 98.45 | 3.45 | 0.05 |
| 1160127 | 6.03 | <0.01 | 85.21 | 95.06 | 2.48 | 0.05 |
| 1160129 | 8.73 | 56.21 | 21.76 | 87.11 | 0.13 | 0.07 |
| 1160130 | 99.64 | <0.01 | 0.01 | 99.47 | 2.55 | 0.06 |
| 1160131 | 70.05 | <0.01 | 7.75 | 86.14 | 2.04 | 0.05 |
| 1160133 | 82.69 | 8.97 | 3.68 | 90.67 | 2.74 | 0.05 |
| 1160134 | 10.03 | 40.41 | 19.23 | 24.50 | 0.06 | 0.07 |
